# The Hippocampal Film Editor: Sensitivity and Specificity to Event Boundaries in Continuous Experience

**DOI:** 10.1523/JNEUROSCI.0524-18.2018

**Published:** 2018-11-21

**Authors:** Aya Ben-Yakov, Richard N. Henson

**Affiliations:** Medical Research Council Cognition and Brain Sciences Unit, University of Cambridge, Cambridge CB2 7EF, United Kingdom

**Keywords:** event boundaries, hippocampus, long-term memory, movie, segmentation

## Abstract

The function of the human hippocampus is normally investigated by experimental manipulation of discrete events. Less is known about what triggers hippocampal activity during more naturalistic, continuous experience. We hypothesized that the hippocampus would be sensitive to the occurrence of event boundaries, that is, moments in time identified by observers as a transition between events. To address this, we analyzed functional MRI data from two groups: one (*n* = 253, 131 female) who viewed an 8.5 min film and another (*n* = 15, 6 female) who viewed a 120 min film. We observed a strong hippocampal response at boundaries defined by independent observers, which was modulated by boundary salience (the number of observers that identified each boundary). In the longer film, there were sufficient boundaries to show that this modulation remained after covarying out a large number of perceptual factors. This hypothesis-driven approach was complemented by a data-driven approach, in which we identified hippocampal events as moments in time with the strongest hippocampal activity. The correspondence between these hippocampal events and event boundaries was highly significant, revealing that the hippocampal response is not only sensitive, but also specific to event boundaries. We conclude that event boundaries play a key role in shaping hippocampal activity during encoding of naturalistic events.

**SIGNIFICANCE STATEMENT** Recent years have seen the field of human neuroscience research transitioning from experiments with simple stimuli to the study of more complex and naturalistic experience. Nonetheless, our understanding of the function of many brain regions, such as the hippocampus, is based primarily on the study of brief, discrete events. As a result, we know little of what triggers hippocampal activity in real-life settings when we are exposed to a continuous stream of information. When does the hippocampus “decide” to respond during the encoding of naturalistic experience? We reveal here that hippocampal activity measured by fMRI during film watching is both sensitive and specific to event boundaries, identifying a potential mechanism whereby event boundaries shape experience by modulation of hippocampal activity.

## Introduction

The hippocampus is perhaps one of the most widely studied regions in the human brain and research has suggested that it has many roles. The role revealed by each study depends on the specific experimental design and comparison of interest. For example, one set of studies may find the hippocampus responds more strongly to subsequently remembered than subsequently forgotten events (Wagner et al., 1998; Kim, 2011), whereas others find it responds more strongly to novel events than previously encountered events (Tulving et al., 1996; Kumaran and Maguire, 2009). The strength of these experiments lies in their ability to probe a specific dimension, comparing the hippocampal response to events in two conditions. However, real life provides us with a continuous stream of complex information—what is an “event” in this context? In other words, what triggers hippocampal activity in naturalistic settings, when we do not present the hippocampus with discretized events?

According to Event Segmentation Theory (Zacks et al., 2007; Kurby and Zacks, 2008), we naturally segment continuous experience into events and this segmentation is driven by moments in time when prediction of the immediate future fails (event boundaries). Segmentation affects not only our perception of the experience, but its subsequent organization in long-term memory (Kurby and Zacks, 2008; Radvansky, 2012; Sargent et al., 2013) such that elements within an event are bound together more cohesively than elements across events (Ezzyat and Davachi, 2011; DuBrow and Davachi, 2013). A natural candidate for mediating the effects of event boundaries on memory is the hippocampus, given multiple findings that together suggest a general sensitivity to prediction error (Strange and Dolan, 2001; Köhler et al., 2005; Kumaran and Maguire, 2006; Axmacher et al., 2010; Chen et al., 2011), combined with its well established role in episodic memory formation (Squire, 1992). Therefore, during naturalistic experience, event boundaries may be expected to be a particularly strong driver of hippocampal activity (but see Schapiro et al., 2016), potentially registering the preceding event to long-term memory as a bound representation (Ben-Yakov and Dudai, 2011; Richmond and Zacks, 2017).

This hypothesis gains further support from studies that have found increased hippocampal activity at the offset of discrete film clips (Ben-Yakov and Dudai, 2011; Ben-Yakov et al., 2013, 2014) or following a context switch (DuBrow and Davachi, 2016) and have related this activity to subsequent memory. However, the stimuli in these studies were clearly dissociated from one another, with boundaries imposed by the experimenter. It is unknown whether the hippocampus responds to subjective event boundaries during continuous, more naturalistic experience. Baldassano et al. (2017) analyzed data from a full-length film and found increased hippocampal responses coinciding with shifts in cortical activity patterns and a coincidence (35–40% match) of pattern shifts and annotated event boundaries. Although these findings hint at a potential link between hippocampal activity and event boundaries, this study did not test a direct link nor the potentially confounding effects of perceptual change.

Here, we examined the direct relationship between hippocampal activity and boundaries and how that activity is modulated by the proportion of observers indicating a boundary (“boundary salience”) after adjusting for various perceptual confounds as well as objective shifts in time/location. Furthermore, even though hippocampal activity may be sensitive to event boundaries, it may not be specific to boundaries. That is, there may be other time points with high hippocampal activity that do not correspond to event boundaries. By defining peaks in hippocampal activity during continuous stimuli, we were able to characterize the specificity of the hippocampal response to event boundaries. Finally, we assessed whether sensitivity to boundary salience was selective to the hippocampus by exploring an atlas of other brain regions.

We addressed these questions by analyzing functional magnetic resonance imaging (fMRI) data from two independent datasets in which participants watched films (Shafto et al., 2014; Hanke et al., 2016). By combining hypothesis-driven and data-driven approaches, we were able to demonstrate that the hippocampal response is sensitive and specific to event boundaries.

## Materials and Methods

### 

#### Data

We analyzed data from two datasets with fMRI scanning of participants viewing films—Stage II of the *Cambridge Centre for Aging and Neuroscience* (Cam-CAN, http://www.cam-can.org) project (for details, see Shafto et al., 2014) and the 3 T audiovisual movie dataset of the *studyforrest* (http://studyforrest.org) project (for details, see Hanke et al., 2016).

#### Participants

##### Cam-CAN.

We used the 253 adults (131 female) who were aged 18–47 (mean age 34.8, SD = 7.9) from the healthy, population-derived cohort tested in Stage II of the Cam-CAN project (Shafto et al., 2014; Taylor et al., 2017). The majority of participants (*n* = 228) were definitively right-handed defined as a handedness measure of ≥50 on a scale of −100 (left) to 100 (right); definitively left-handed were defined as ≤−50; and those with a handedness measure of −49 to 49 were considered undetermined. All participants were native English speakers. Ethical approval was obtained from the Cambridgeshire Research Ethics Committee and all participants gave their written informed consent before participation.

##### studyforrest.

The current analysis focused on 15 participants (mean age 29.4, range 21–39, 6 female) for whom fMRI was collected during film viewing (Hanke et al., 2016; Sengupta et al., 2016). The participants were all right-handed native German speakers with normal visual function. Ethical approval was obtained from the Ethics Committee of the Otto-von-Guericke University and all participants gave informed consent before participation.

#### Experimental design

##### Cam-CAN.

Participants viewed an abridged version of Alfred Hitchcock's black-and-white television drama “Bang! You're Dead” (Hasson et al., 2008, 2010) edited from 30 min to 8 min while maintaining the plot (Shafto et al., 2014). The film was chosen to be compelling but unfamiliar to participants.

##### studyforrest.

Participants viewed the film “Forrest Gump” (R. Zemeckis, Paramount Pictures, 1994) with German dubbing. The film was edited to be 2 h and divided into 8 segments, each ∼15 min long, presented in a separate run (Hanke et al., 2016). All participants except one had previously seen the film (and the additional participant had previously heard an audio-only version).

##### Film segmentation.

We identified the occurrence of event boundaries using subjective annotations, recorded using PsychoPy version 1.85.0 (Peirce, 2007). Sixteen observers viewed each of the films and indicated with a keypress when they felt “one event (meaningful unit) ended and another began” (based on the event segmentation approach in Newtson, 1973; Zacks et al., 2010). In terms of granularity of segmentation (coarse/fine-grained), participants were instructed to segment in the manner that felt most natural to them. Eight of the 16 observers watched “Forrest Gump” with German dubbing (as in *studyforrest*) and eight watched the film in English. To account for response time, 0.9 s was subtracted from the logged button presses (calculated based on prior testing to estimate reaction time). If different observers marked boundaries within 1 TR (repetition time, TR ∼2 s; see below) of one another, then these were treated as a single boundary (and the time of the boundary was defined as the average time identified by the different observers). Because events in close proximity (relative to the sampling resolution) are not properly separable, we ran an additional iteration to combine boundaries that were <2 TRs apart. All boundaries that had been identified by at least 2 observers and were within 2 TRs of one another were then replaced by a single boundary (the time of the boundary was defined as the average of all observers in the 2 original boundaries and the number of observers was set to be a summation of the 2 original ones). The process was run in two iterations (limiting the second iteration only to boundaries identified by at least two observers) rather than initially combining all boundaries separated by 2 TRs because the latter would have resulted in long chains such that 2 boundaries separated by over 12 s could end up being averaged together. Boundaries within 10 s of the end of the run were also removed because they could not be properly estimated in the single-event analysis. To avoid including spurious boundaries (e.g., due to accidental button presses), we set a threshold for the number of observers required to identify a boundary in order for it to be included in the analysis. We estimated the “true” number of boundaries by averaging the total number of boundaries across observers and set the threshold to be the one that yielded the closest number of boundaries to this estimation. In both studies, the optimal threshold was found to be five, so only boundaries identified by a minimum of five observers were included in the analysis. In Cam-CAN, the final set of boundaries consisted of 19 boundaries separated by 6.5–93.7 s (mean 23.9 s, SD = 21.8 s) and a range of 5–16 observers who identified each boundary (5 observers: 3 boundaries; 6:4; 8:1; 10:2; 12:2; 13:2; 14:2; 15:2; 16:1). In *studyforrest*, there were 161 boundaries (12–25 per run) separated by 4.9–167.7 s (mean 43.3 s, SD = 33.6 s) and a range of 5–15 observers who identified each boundary (5 observers: 37 boundaries; 6:26; 7:16; 8:13; 9:15; 10:18; 11:12; 12:9; 13:9; 14:5; 15:1). The boundaries were divided into three levels of salience (according to the number of observers that identified the boundary) such that each level contained approximately the same number of events. In Cam-CAN, there were 7 low salience events (5–6 observers), 5 medium salience events (7–12 observers) and 7 high salience events (13–16 observers). In *studyforrest*, there were 60/43/54 low/med/high salience events (low = 5–6; med = 7–9; high = 10–16). An advantage of using an independent set of observers for segmentation is that asking participants to indicate boundaries while watching a film in the scanner may alter the brain responses, for example, by making boundaries task relevant and no longer corresponding to naturalistic viewing. An alternative would be to ask each participant to annotate the film again after having watched it in the scanner, but a concern of particular relevance for the hippocampus is that memory for the film might affect the decisions about event boundaries.

To determine whether watching “Forrest Gump” in English or German would affect segmentation, we ran the procedure described above for identifying boundaries separately for the subgroups that watched the film in each language. We compared the number of matching boundaries (up to 1 TR apart) between the two groups (*n* = 77) with the number of matching boundaries when randomly dividing the observers into two groups (mean = 78.4). Because there was no significant difference between the groups (46% of random divisions yielded a lower match), we combined the two language groups for all subsequent analyses.

Because familiarity has been shown to affect the exact timing of boundary-related activity (Baldassano et al., 2017), we assessed the familiarity of our observers relative to the familiarity of the original participants. Although familiarity was not recorded in Cam-CAN, the film was chosen to be unfamiliar to participants. Of the 16 observers who segmented the Cam-CAN film, only one had previously seen bits and pieces of the film and it was unfamiliar to the rest. Therefore, both fMRI participants and observers were generally unfamiliar with the Cam-CAN film. In *studyforrest*, the majority of participants (14/15) had previously seen the film and had varying degrees of familiarity. Although we could not match the exact degree of familiarity among the observers who segmented the film, the majority had also previously seen “Forrest Gump” (13 had seen it, two had not, and familiarity information was not collected for an additional observer). Therefore, both fMRI participants and observers were generally familiar with the *studyforrest* film.

#### fMRI acquisition

##### Cam-CAN.

Imaging was performed on a 3 T Siemens TIM Trio scanner at the Medical Research Council Cognition and Brain Sciences Unit using a 32-channel head coil. High-resolution 3D T1-weighted structural images were acquired using a magnetization prepared rapid acquisition gradient-echo (MP-RAGE) pulse sequence (1 × 1 × 1 mm resolution, TR = 2250 ms, TE = 2.99 ms, TI = 900 ms, flip angle = 9°, FOV = 256 × 240 × 192 mm, GRAPPA acceleration factor = 2). Functional images were acquired using a multiecho, T2*-weighted echoplanar imaging (EPI) sequence [TR = 2470 ms, TE (5 echoes = 9.4, 21.2, 33, 45, 57 ms), flip angle = 78°, 32 axial slices with 3.7 mm thickness and a 20% gap, FOV = 192 × 192 mm, voxel size = 3 × 3 × 4.44 mm].

##### studyforrest.

Imaging was performed on a 3 T Achieva scanner (Philips Medical Systems) using a 32-channel head coil. High-resolution T1-weighted structural images (Hanke et al., 2014) were acquired using a 3D turbo field echo sequence [acquisition voxel size of 0.7 mm with a 384 × 384 in-plane reconstruction matrix (0.67 mm isotropic resolution), TR = 2500 ms, TE = 5.7 ms, TI = 900 ms, flip angle = 8°, FOV = 191.8 × 256 × 256 mm, bandwidth 144.4 Hz/px, sense reduction AP 1.2, RL 2.0]. Functional images (Hanke et al., 2016) were acquired using a gradient-echo, T2*-weighted EPI sequence (TR = 2000 ms, TE = 30 ms, flip angle = 90°, 35 axial slices with 3.0 mm thickness and a 10% gap, FOV = 240 × 240 mm, voxel size = 3 × 3×3 mm). Slices were automatically positioned in AC–PC orientation using SmartExam (Philips) such that the topmost slice was at the superior edge of the brain.

#### Data preprocessing

Data from both studies were preprocessed using SPM12 (http://www.fil.ion.ucl.ac.uk/spm), automated with the Automatic Analysis (AA) 4.2 pipeline system (Cusack et al., 2014; for details on the specific analysis used, see Taylor et al., 2017) in MATLAB (version 8.5.0 R2015a, The MathWorks). T1 anatomical images were coregistered to the Montreal Neurological Institute (MNI) template using rigid body transformation, bias corrected, and segmented by tissue class. Diffeomorphic registration was then applied to the gray matter to create a group template, which was in turn affine transformed to MNI space. For the Cam-CAN functional images, the data from the multiple echos were first averaged, weighted by the contrast-to-noise ratio of each echo for each voxel. For both datasets, the functional images were corrected for motion and then corrected for slice acquisition times by interpolating to the middle slice. The images were rigid-body coregistered to the corresponding T1 image and the spatial transformations from that image to MNI space (diffeomorphic + affine) were applied. Finally, effects of abrupt motion were reduced by applying wavelet despiking (Patel et al., 2014). In the Cam-CAN data, two additional steps were performed: anatomical segmentation was informed by additional T2 images and field maps were used to correct EPI distortions before motion correction (no T2 images or field maps were available for the *studyforrest* data). High-pass filtering (cutoff of 256 s) was implemented with a cosine basis set as part of the general linear model (GLM) within SPM12 (for the pattern analyses, the data were filtered first by taking the residuals from a GLM containing just the cosine terms).

#### Anatomical ROI definition

After alignment to the group template in MNI space, T1 images were averaged separately for each dataset. Bilateral group ROIs of the hippocampus were then manually traced using ITK-SNAP (http://www.itksnap.org, Yushkevich et al., 2006). Because right/left and anterior/posterior hippocampus yielded similar results in the effects of interest (and we did not have an a priori hypothesis regarding such differences), the analyses were all run on the average across left and right hippocampus. The visual (“visual cortex V1”), auditory (“primary auditory cortex TE1.0 + TE1.1”), and angular gyrus ROIs were defined using the Juelich atlas (Eickhoff et al., 2005). The angular gyrus ROI was defined using the same ROI as in Baldassano et al. (2017). To run the whole-brain analysis using the same mixed-effects models used for the hippocampal analysis, we used an ROI-based approach, running the model on all ROIs from the Harvard–Oxford Atlas (Desikan et al., 2006).

#### Angular gyrus (AG) pattern shift analysis

In a recent study, Baldassano et al. (2017) used a data-driven approach for event segmentation of realistic experience. They found that, in high-level regions such as AG and posterior medial cortex, points in time of rapid change in activity patterns (cortical event boundaries) corresponded to event boundaries as defined by human observers. Moreover, cortical event boundaries, particularly in the AG, coincided with an increase in hippocampal univariate activity. Our analysis focused on the AG using an anatomical ROI from the Eickhoff atlas (Eickhoff et al., 2005). To test whether AG pattern changes could account for any hippocampal response to event boundaries in the current studies, we defined AG boundaries implementing the hidden Markov model segmentation method from Baldassano et al. (2017) with one alteration. In the original segmentation procedure, the number of events was estimated in a data-driven manner. In the current studies, this approach did not yield optimal results. In *studyforrest*, the estimated number of events ranged from 4 to 59 per run, whereas the actual number of boundaries ranged from 16 to 25, so we defined the number of events in each scan according to the number of event boundaries. We classified the AG pattern shifts according to whether they matched an event boundary (i.e., occurred up to 2 TRs after) and separately averaged the hippocampal response around match/non-match AG pattern shifts. In the AG analysis, we defined a match as up to 2 TRs (4.94/4 s in Cam-CAN/*studyforrest*), as opposed to 1 TR for the hippocampal analyses, to be consistent with the original analysis in Baldassano et al. (2017), which defined a match as up to 3 TRs that equaled 4.5 s in their study. The hippocampal response was calculated by *z*-scoring the entire time course for each run of each participant and then averaging over participants, over left/right hippocampus, and over event boundaries. An examination of the original results (Baldassano et al., 2017) reveals that the peak hippocampal response occurred in the first 4–5 s after the AG shift. Therefore, we computed the amplitude of the hippocampal response as an average of TRs 0–2 (0–4.94 s in Cam-CAN, 0–4 s in *studyforrest*) relative to the AG shift.

#### Statistical analysis

##### Assessing significance of response to boundaries.

**Figure 1.**
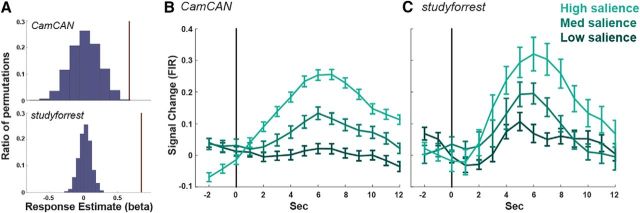
Hippocampal response to event boundaries. ***A***, Average amplitude of the canonical response to an event boundary (brown lines) relative to the distribution of responses when randomly shuffling event order shown for Cam-CAN (top) and *studyforrest* (bottom). ***B***, ***C***, Average response across participants to event boundaries binned by boundary salience in Cam-CAN (***B***) and *studyforrest* (***C***). The per-participant time course was calculated using an FIR and error bars indicate the SEM at each time point. The vertical black line represents the event boundary.

The amplitude of the hippocampal response to event boundaries was measured using a GLM with a single predictor for all event boundaries [i.e., a stick function at event boundaries, convolved with the canonical hemodynamic response function (HRF)], together with the high-pass filter regressors as nuisance predictors. To assess the significance of the hippocampal response to boundaries, we compared it with the measured response to boundaries in 1000 random permutations of the event durations (the intervals between consecutive boundaries) and used the ratio of permutations with a larger response than the intact one (in absolute value for a two-tailed estimation) to derive a *p*-value (Fig. 1*A*). A similar approach was used to assess the significance of the hippocampal response at AG pattern shifts, comparing the amplitude of the response to the amplitude calculated when permuting events (here defined as the epochs between AG pattern shifts).

##### Mixed-effects model.

When using films as memoranda, the stimulus-as-fixed-effects fallacy (Clark, 1973; Westfall et al., 2016) becomes more pertinent because each film has specific characteristics that do no generalize to all films. We thus used a mixed-model for statistical analysis, incorporating both participants and items (event boundaries) as random effects (Baayen et al., 2008). We first estimated the hippocampal response to each participant-by-item in a GLM with a separate predictor for each boundary of each participant because well as high-pass filter confound predictors. For the purposes of this analysis, all boundaries closer than 6 s to one another were removed because the responses to events in such close proximity cannot be dissociated in a single-trial GLM. This did not change the number of event boundaries in Cam-CAN (19), but reduced the number from 161 to 157 in *studyforrest*. The resulting betas were then submitted to two linear mixed-models, one with boundary salience as the (fixed) effect of interest based on the number of observers who identified the item as a boundary (divided into three bins of low/medium/high salience with approximately equal numbers of events in each) and the second with nObservers as the (fixed) effect of interest (the precise number of observers who identified the boundary, with no binning). Both models incorporated participant and item as random effects. We tested a linear dependence between hippocampal activity and salience/nObservers as we did not have an a priori reason to expect a nonlinear dependence. However, significant results in these models do not necessarily indicate that the nature of dependence is strictly linear. In *studyforrest*, there was an additional fixed effect of run number for each of the eight runs. Although *studyforrest* included only right-handed participants and Cam-CAN included both right- and left-handed participants, we did not observe an effect of handedness in Cam-CAN. We thus included all participants in the Cam-CAN analysis regardless of handedness.

The linear models were then fitted using restricted maximum likelihood with the lme4 (Schielzeth and Nakagawa, 2013) and lmerTest (Kuznetsova et al., 2017) packages in R (R 3.1.3, R Core Team, 2017, https://www.R-project.org/), with the following formulas:

Cam-CAN: ′betas ∼ salience/nObservers + (1|participant) + (1|event boundary)′

*studyforrest*: ′betas ∼ salience/nObservers + runNum + (1|participant) + (1|event boundary).

Where (1|*x*) indicates a random effect, and salience/nObservers indicates that either salience or nObservers was used in the model.

Because event boundaries are typically characterized by various types of visual and auditory change, we ran a second analysis incorporating multiple predictors estimating the following attributes (a brief summary is followed by a more detailed description of each predictor, including its correlation with nObservers):

(1) isLoc/isTemp: shift in location/time; (2) visDist: visual distance between frames immediately preceding and frames immediately following a boundary; (3) visCorr: visual correlation between frames immediately preceding and frames immediately following a boundary; (4) visHistDist: the distance between the color histograms of the frames before and after a boundary; (5) lumdist: difference in overall luminance before and after a boundary; (6) DCNN: the correlation between the layers of a deep neural net run on the frames before and after a boundary; (7) psdCorr: correlation of the power spectral density (PSD) before and after a boundary as a measure of auditory similarity; (8) psdDist: the distance of the PSD across a boundary; (9) absVolDist: absolute difference in volume across a boundary; (10) V1 and A1 betas: average V1/A1 response (across participants) to each event; and (11) isAG: binary predictor indicating whether a boundary coincides with a pattern shift in the angular gyrus.

##### isLoc/isTemp.

Event boundaries are often characterized by a change in location, time, or both. Due to the sensitivity of the hippocampus to both time and space, we added predictors to account for these changes. For the Cam-CAN film, location/temporal changes were identified by the authors and incorporated into a single predictor, isLocTemp (because the two always coincided). The correlation of isLocTemp with nObservers was 0.8 (*p* = 4 × 10^−5^). The *studyforrest* project already includes annotations for every film shot, including the spatial location and indication of temporal progression relative to the preceding shot (Häusler and Hanke, 2016). These were used to create separate predictors for change in location (isLoc) or time (isTemp). The correlation of isLoc/isTemp with nObservers was 0.42/0.47 (*p* = 3.2 × 10^−8^/*p* = 5.3 × 10^−10^). The correlation between isLoc and isTemp was 0.81 (*p* = 3.9 × 10^−37^).

##### visDist.

The visual distance between each pair of frames (up to 1500 frames/1 min apart) was calculated using IMage Euclidean distance (IMED, Wang et al., 2005). This measure takes into account the similarity, not only with the parallel pixel in the second image, but also similarity with surrounding pixels (weighted by a distance function), and is thus less sensitive to small movements between frames. Due to computational memory restrictions, we first resized the images to 1/8 of the original resolution (resulting in 96 × 72 pixels for Cam-CAN frames and 192 × 82 pixels for *studyforrest* frames) and then compared each pair of frames with the same parameters (distance weighting matrix G and width parameter σ) used in Wang et al. (2005) with one exception: For ease of calculation, only pixels in a 9 × 9 square around a given pixel were taken into consideration because beyond this range the weights in the G matrix were virtually zero. The original distance measure is for grayscale images, summing the distance of all pixels to calculate the global image distance. To extend the measure for color images (in *studyforrest*), we calculated the distance for each channel of each pixel separately and then summed over channels and pixels for the global measure.

After having calculated the visual distance between pairs of frames, the distance across each boundary was defined as the maximal distance between any frame in the 1 s window before the boundary with any frame in the 1 s window following the boundary. The same approach to calculation visual change across boundaries was applied to all following visual measures. Correlation with nObservers was 0.31/0.07 (*p* = 0.2/0.38) in Cam-CAN/*studyforrest*.

##### visCorr.

The visual correlation between each pair of frames (up to 1500 frames/1 min apart) was calculated using IMage Normalized Cross-Correlation (IMNCC, Nakhmani and Tannenbaum, 2013). This measure uses a similar approach to IMED for calculating correlations while taking into account spatial relationships of pixels. When calculating IMNCC, we used the same parameters (G, σ) as for the IMED calculation above. Correlation with nObservers was −0.31/−0.15 (*p* = 0.19/0.05) in Cam-CAN/*studyforrest*.

##### visHistDist.

In addition to measuring the visual distance between frames, we measured the visual distance between the histograms of the frames. The rationale for this is that two frames may be quite distinct in terms of the objects that they contain and their spatial layout, but still depict a similar setting with similar lighting and colors. To test for such global similarity, we calculated the histogram of each frames and computed the Euclidean distance between the histograms. For RGB frames (*studyforrest*), we calculated the histogram for each channel separately and then computed the distance over all bins of the three histograms together. Correlation with nObservers was 0.3/0.12 (*p* = 0.21/0.12) in Cam-CAN/*studyforrest*.

##### lumDist.

To detect global lighting changes, we calculated the difference in global luminance between frames, first calculating the average luminance over all pixels and then taking the absolute difference. Correlation with nObservers was 0.27/−0.02 (*p* = 0.26/0.77) in Cam-CAN/*studyforrest*.

##### DCNN.

DCNNs are able to extract higher-order information from images beyond the low-level perceptual properties, with higher layers corresponding to higher-order visual regions (Güçlü and van Gerven, 2015). To automatically identify similarities between frames at multiple levels of visual feature hierarchy, we submitted each frame to AlexNet, one of the most commonly used DCNNs for image identification (Krizhevsky et al., 2012). We then correlated the representation of each pair of frames in each layer of the network. Calculation of the correlation across boundaries was identical to the rest of the visual features, with the exception that every boundary had 21 correlation values, one for each layer of the network, yielding 21 vectors of per-boundary correlations. Because these vectors were highly correlated, we ran singular value decomposition (SVD) on the correlation matrix and used the set of first components that explained 90% of the variance in each study (6 in Cam-CAN and 7 in *studyforrest*). Correlation with nObservers ranged from −0.53 to 0.45 in Cam-CAN (*p* = 0.02–0.68) and from −0.15 to 0.2 (*p* = 0.01–1) in *studyforrest*.

##### psdCorr.

To assess the auditory difference across boundaries, we calculated the PSD in the 500 ms epochs before and after each boundary with a cutoff frequency of 5000 Hz (when examining the entire audio, >99% of the power was below this cutoff). The preboundary and postboundary PSDs were correlated as a measure of auditory similarity. Correlation with nObservers was −0.27/−0.07 (*p* = 0.26/0.33) in Cam-CAN/*studyforrest*.

##### psdDist.

In addition to measuring the audio-correlations, we calculated the Euclidean distance between the PSDs. Correlation with nObservers was 0.24/−0.03 (*p* = 0.33/0.68) in Cam-CAN/*studyforrest*.

##### absVolDiff.

To detect abrupt volume changes, we calculated the average volume in the 100 ms before and after the boundary, taking the absolute difference as the measure of volume change. Correlation with nObservers was 0.12/−0.01 (*p* = 0.62/0.88) in Cam-CAN/*studyforrest*.

##### V1Betas, A1Betas.

To account for additional low-level visual/auditory changes that may not be captured by the stimulus-defined predictors, we added the per-trial activity in V1 and A1. The per-trial response in these regions was calculated in a single-trial GLM and averaged over participants to extract the stimulus-driven component of their activity. Correlation of V1Betas with nObservers: 0.56/0.28 (*p* = 0.01/0.0005) in Cam-CAN/*studyforrest* and for A1Betas: 0.35/0.25 (*p* = 0.14/0.002).

##### isAG.

To account for the effect of AG pattern shifts, we classified the event boundaries according to whether they matched an AG pattern shift (occurring up to 2 TRs before it) and added this as a binary predictor. Correlation with nObservers was 0.56/0.06 (*p* = 0.01/0.46) in Cam-CAN/*studyforrest*.

Adding all predictors to the model in Cam-CAN was not possible because we would have 19 predictors for 19 events. We thus ran separate SVDs on the visual, auditory and higher-order visual (CNN) predictors, taking the two first components in each. The models for the two experiments were then fit using the following formulas:

Cam-CAN: ′betas ∼ salience/nObservers + isLocTemp + visComp1 + visComp2 + audComp1 + audComp2 + DCNN1 + DCNN2 + isAG + (1|participant) + (1|event boundary)′

*studyforrest*: ′betas ∼ salience/nObservers + runNum + isLoc + isTemp + visDist + visCorr + visHistDist + lumDiff + DCNN[1…7] + psdCorr + psdDist + absVolDiff + V1Betas + A1Betas + isAG + (1|participant) + (1|event boundary)′

Significance of the predictor of interest (salience/nObservers) was estimated using the ANOVA function of the lmerTest package (Kuznetsova et al., 2017), with type III error calculation and the Satterthwaite approximation for degrees of freedom (Satterthwaite, 1941), found to yield optimal *p*-value estimations for mixed models (Luke, 2017). The effect size (marginal *R*^2^) was calculated using the r.squaredGLMM function of the MuMIn package (MuMIn: Multi-Model Inference. R package version 1.15.6, https://CRAN.R-project.org/package=MuMIn; see Nakagawa and Schielzeth, 2013 for a discussion of this approach).

#### Plotting

**Figure 2.**
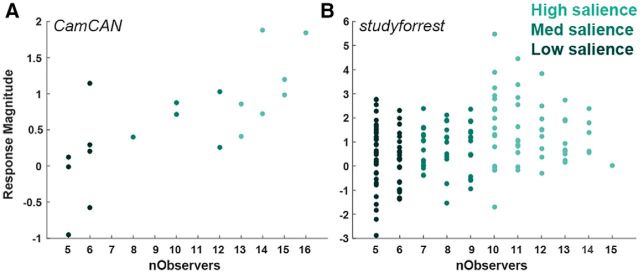
Sensitivity of hippocampal response to boundary salience. The average magnitude of the canonical response to event boundaries (averaged over participants) by the number of observers that marked them (nObservers). Each dot represents the average response to a single boundary and its color reflects its salience level. Results are presented for Cam-CAN (***A***) and *studyforrest* (***B***).

The time course of the average response to each condition (Fig. 1*B*,*C*) was calculated using an FIR analysis of the hippocampal ROI. We extracted and normalized (*z*-score) the time course from the hippocampal ROI and then interpolated the time course to be in 1 s resolution for both projects. Event boundaries were binned according to the number of observers who identified them into low/medium/high levels of salience (see “Film segmentation” section). We constructed a GLM with a separate predictor for each condition X time-point in the range [−2… 12 s] relative to stimulus onset at time 0. This yielded an estimate of the per-condition response for each participant, which was used for plotting purposes (Figure 1*B*,*C*). In Figure 2, the hippocampal response is plotted against the number of observers, with each dot representing the average response to each boundary (averaged across participants). The FIR analysis is participant based, whereas the scatter plot in Figure 2 is item based. Both types of averaging are used for illustration purposes only (with mixed effects used for statistical analysis).

#### Hippocampal data-driven segmentation

In addition to the hypothesis-driven approach, we defined hippocampal events as the set of momentary events that, when modeled, minimized the residual error in the hippocampal time course. The raw hippocampal time course of each participant (averaged over all voxels in the hippocampus) was first high-pass filtered and *z*-scored and then averaged across participants. The number of hippocampal events was predefined as the number of event boundaries and we iteratively chose hippocampal events until this number was reached (in *studyforrest*, the number of event boundaries was calculated per-run). Hippocampal events were chosen in the following manner, starting with a GLM (*M*) that included only a constant predictor and an empty list of hippocampal events:

1. Run over all TRs that have not yet been added to the model.
2. For each TR, create a temporary model *M*_temp_ by adding a predictor with a stick function at that TR, convolved with the canonical HRF, and calculate the residual sum of squares (RSS) of this model.
3. Choose the TR (model) that most reduces the RSS and define it as a hippocampal event, adding its corresponding predictor to *M* before the next iteration. This was done under two constraints: -the β value associated with the hippocampal event is positive and does not flip the signs of any β values of the hippocampal events defined in previous iterations.

A hippocampal event (data-driven) was considered to match an event boundary (hypothesis-driven) if it was up to 1 TR from a boundary. To calculate the significance of the number of matching events, we compared it with the number of matches when randomly shuffling the subjective events 1000 times (while maintaining the event durations).

## Results

Our main question of interest was whether subjective event boundaries trigger hippocampal activity during continuous naturalistic experience. This entails estimating both the sensitivity and specificity of hippocampal activity to event boundaries. Sensitivity was assessed in a hypothesis-driven approach, by examining the hippocampal response at event boundaries subjectively annotated by independent observers. Specificity was assessed using a data-driven approach, identifying hippocampal events based on the amplitude of the hippocampal response, and testing the overlap between these events and subjective boundaries.

### Hippocampus is sensitive to event boundaries

In the hypothesis-driven analysis, we defined event boundaries by using a separate group of 16 people who indicated with a keypress when they experienced an event shift while watching each film (see Materials and Methods). We first assessed overall sensitivity to the occurrence of event boundaries by comparing the estimated response to a boundary with the distribution of estimated responses when shuffling the events (the durations between boundaries). In Cam-CAN, this yielded a *p*-value of 0.002 and, in *studyforrest*, the estimated response to boundaries in the intact event order was larger than for any of the random permutations (equivalent to *p* < 0.001, Fig. 1*A*). Having revealed overall sensitivity to boundaries, we set out to determine whether the hippocampal response was modulated by “boundary salience,” estimated by the number of observers who marked the boundary. For illustration purposes, we first plotted the average response to each boundary (averaged across participants) by the number of observers that identified the boundary. In both studies, we found an overall higher response when a larger number of observers marked a moment in time as a boundary (Fig. 1*B*,*C*). To quantify this effect, we ran two mixed-effects models. Because we did not necessarily anticipate a linear correspondence between the precise number of observers and hippocampal activity, the first model estimated sensitivity to boundary salience by binning boundaries into low/medium/high salience approximately equated for the number of boundaries in each bin. Then, in a second model, we additionally tested whether there existed a linear relationship between hippocampal activity and the number of observers. Within each model, salience or nObservers was the (fixed) effect of interest and participant and event boundaries were random effects as reported below for each film separately.

### Cam-CAN

We found an increase in the hippocampal response to a boundary as a function of the boundary salience (Fig. 2*A*) and the mixed-effects model revealed that this modulation was significant (*p* = 0.0003, *F*_(1,17)_ = 20.12). The effect size was small (*R*^2^ = 0.04), but it is worth noting that the model was run on single trial estimates, so effect sizes are expected to be small compared with models which average over participants or within-condition items. We observed significant modulation, not only by the coarse measure of boundary salience, but also by the number of observers who marked each boundary (*p* = 0.0001, *F*_(1,17)_ = 23.7, *R*^2^ = 0.04).

Because event boundaries are typically characterized by visual and auditory shifts, we fit an additional model to test whether low-level visual and auditory changes could account for the hippocampal sensitivity to boundaries. These included various measures of visual and auditory change across the boundary, such as luminance and sound-level differences as well as responses extracted from early visual and auditory cortices (for a full list, see Materials and Methods). In addition, we added predictors to account for two additional hypotheses regarding the trigger for hippocampal responses. First, a recent study (Baldassano et al., 2017) found increased hippocampal activity following boundaries defined by cortical pattern shifts, particularly in the AG. These cortical pattern changes exhibited a large degree of overlap with subjectively annotated boundaries, so hippocampal sensitivity to event boundaries could arise from AG pattern shifts. To test this, we added to the model a binary predictor indicating whether an AG boundary occurred in temporal proximity to each annotated one. Second, we added a predictor of objective shift in time/location (isLocTemp) to determine whether the sensitivity to boundaries was driven by such changes (see Materials and Methods for definition). When adding all predictors, the effects of boundary salience and nObservers were no longer significant (*p* = 0.94, *F*_(1,7)_ = 0.007 and *p* = 0.88, *F*_(1,7)_ = 0.02, respectively), potentially due to the large number of predictors (*n* = 11) relative to the number of event boundaries (*n* = 19). Indeed, when adding each covariate to the model separately, both the effect of salience and the effect nObservers remained significant (Table 1). Most event boundaries were characterized by a change in location such that the predictors for isLocTemp and nObservers were highly dependent (*r* = 0.8) and their respective contributions could not be properly dissociated. Therefore, we cannot conclude, based on this dataset alone, whether this modulation by boundary salience may be accounted for by other perceptual factors, particularly spatial/temporal change.

**Table 1.** Significance of mixed-effects models when separately accounting for each covariate

Predictor	Salience		nObservers	
	*F*_(1,16)_	*p*	*F*_(1,16)_	*p*
isLocTemp	4.9	0.04	5.7	0.03
visDist	14.3	0.002	18.7	0.0005
visCorr	18.3	0.0006	21.5	0.0003
visHistDist	16.5	0.0009	19.7	0.0004
lumDiff	17.2	0.0008	21.8	0.0003
DCNN[1… 6]	minF = 12.8	maxP = 0.003	minF = 15.6	maxP = 0.001
psdCorr	17	0.0008	20.1	0.0004
psdDist	18.6	0.0005	22.5	0.0002
absVolDiff	18.6	0.0005	22	0.0003
V1Betas	9.8	0.006	11.9	0.003
A1Betas	16.8	0.0008	19.5	0.0004
isAG	16.9	0.0008	19.7	0.0004

Shown are the results of the mixed-effects models separately accounting for each of the perceptual confounds and the shifts in time/location. Results are presented both for models with salience as the effect of interest and for models with nObservers as the effect of interest. All models were fitted using the following formula template (replacing <covariate> with each of the potential predictors): betas ∼ <covariate> + salience/nObservers + (1 participant) + (1 event boundary).

### studyforrest

In *studyforrest*, we similarly found an increase with boundary salience (Fig. 2*B*, boundary salience: *p* = 2.2 × 10^−6^, *F*_(1,148)_ = 24.3, *R*^2^ = 0.02; nObservers: *p* = 3.6 × 10^−5^, *F*_(1,148)_ = 18.2, *R*^2^ = 0.01). Now, however, even when adding all confounds as well as separate predictors for spatial and temporal changes (isLoc, isTemp), the boundary effects remained significant (boundary salience: *p* = 0.002, *F*_(1,129)_ = 9.5; nObservers: *p* = 0.03, *F*_(1,129)_ = 4.9). The hippocampus also demonstrated sensitivity to change in location (*p* = 0.003, *F*_(1,148)_ = 8.8) or time (*p* = 0.002, *F*_(1,148)_ = 9.4), but not when accounting for perceptual confounds, boundary salience, or nObservers (minimal *p*-value in these tests = 0.18, *F*_(1,147)_ = 1.5). Therefore, whereas changes in location/time modulate hippocampal activity, this does not seem to account for its sensitivity to boundary salience. Moreover, in neither study was change in time/location a significant predictor of hippocampal activity after accounting for salience.

Therefore, in both studies, we found that the hippocampus is sensitive to boundary salience and to the specific number of observers who subjectively reported a shift. Furthermore, in *studyforrest*, where there were a sufficient number of event boundaries to assess the relative contribution of potential additional drivers, we found that the sensitivity to the number of observers could not be explained by objective measures such as visual/auditory change or by change in location/time. The modulation of hippocampal response by boundary salience suggests the hippocampus is not only sensitive to the occurrence of event boundaries, but also to their salience.

### Both hippocampal activity and AG patterns are driven by event boundaries

**Figure 3.**
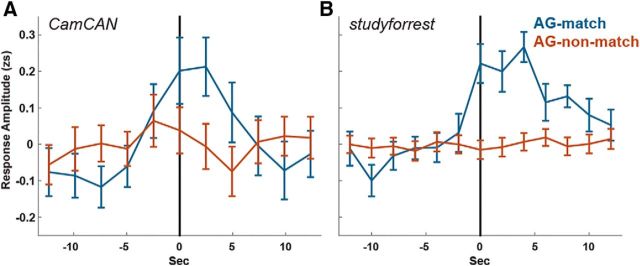
Hippocampal response to AG pattern shifts. Average zscored (zs) hippocampal response at AG pattern shifts that match/do not match annotated event boundaries. Time 0 (vertical lines) represents the time of the pattern shift uncorrected for hemodynamic delay. Error bars indicate SEM (across pattern shifts). Results are shown for Cam-CAN (***A***) and *studyforrest* (***B***).

Another potential explanation for the increase in hippocampal activity at event boundaries is that event boundaries elicit cortical pattern shifts, which in turn drive hippocampal activity (Baldassano et al., 2017). Although inclusion of the AG predictor did not account for the sensitivity to boundary salience in the above analyses, AG pattern shifts could still account for the overall hippocampal response to boundaries regardless of their boundary salience. To test this, we divided AG pattern shifts into those that corresponded with an event boundary (AG match) and those that did not (AG non-match), averaging the hippocampal response around each type (Fig. 3). We replicated the finding of Baldassano et al. (2017) of an increased hippocampal response to overall AG pattern shifts (Cam-CAN: *p* = 0.04, *studyforrest*: *p* < 0.001, higher than all random permutations). However, this increase was only found for AG pattern shifts that coincided with event boundaries: For matching shifts (9/19 in Cam-CAN and 35/161 in *studyforrest*), there was a significant increase in hippocampal activity at the shift (Cam-CAN: *p* = 0.007, *studyforrest*: *p* < 0.001), whereas for the non-matching shifts, there was no significant increase in hippocampal activity (Cam-CAN: *p* = 0.97, *studyforrest*: *p* = 0.74). In a direct comparison of match and non-match shifts, the difference was significant in *studyforrest* (Cam-CAN: *p* = 0.08, *studyforrest*: *p* < 0.001). This suggests that both AG pattern shifts and increased hippocampal activity are driven by the occurrence of event boundaries rather than AG pattern changes and hippocampal activity being directly related.

### Selectivity of hippocampal modulation by boundary salience

Our a priori interest, based on previous studies, was the effect of event boundaries on hippocampal activity. However, additional regions may show similar modulation by boundary salience given the large number of regions that have been reported to respond to event boundaries in general (Zacks et al., 2001, 2010). We tested this by rerunning the above mixed models on anatomically defined regions across the brain taken from the Harvard–Oxford Atlas (Desikan et al., 2006). We averaged across left and right homologous regions. Of the 55 homologous regions in the atlas, five showed a significant modulation by boundary salience (when correcting for multiple comparisons using Holm–Bonferroni) in both experiments (Table 2): the hippocampus, posterior cingulate cortex (PCC), precuneus, posterior parahippocampal cortex, and lingual gyrus. Of these, the effect remained significant only in the hippocampus and PCC when adding the perceptual and objective shift predictors in *studyforrest* (with a trend in precuneus, *p* = 0.06). When testing modulation by nObservers, only hippocampus and PCC were significant in both experiments (Table 2) and the effect again remained significant in both regions when accounting for the additional covariates. Therefore, although several regions demonstrate sensitivity to boundary salience, only in the hippocampus and PCC can this sensitivity not be accounted for by perceptual confounds or objective shifts in time/location.

**Table 2.** Regions demonstrating a significant modulation by boundary salience

Regions modulated by salience	Cam-CAN			*studyforrest*			*studyforrest* with covariates	
	*F*_(1,17)_	*p*	*R*^2^	*F*_(1,148)_	*p*	*R*^2^	*F*_(1,129)_	*p*
Hippocampus	18.6	0.0005	0.04	23.4	3.3 × 10^−6^	0.02	9.1	0.003
Cingulate gyrus, posterior division	30.5	3.7 × 10^−5^	0.1	25	1.6 × 10^−6^	0.02	8.3	0.005
Precuneous cortex	22.6	0.0002	0.14	11.2	0.001	0.01	3.6	0.06
Parahippocampal gyrus, posterior division	16.1	0.0009	0.09	14	0.0003	0.01	1.1	0.3
Lingual gyrus	17.5	0.0006	0.16	15	0.0002	0.03	0.02	0.89

Regions modulated by nObservers	*F*_(1,17)_	*p*		*F*_(1,148)_	*p*		*F*_(1,129)_	*p*
Hippocampus	18.6	0.0002	0.04	17.7	4.5 × 10^−5^	0.01	4.7	0.03
Cingulate gyrus, posterior division	30.5	6.6 × 10^−5^	0.1	20.3	1.3 × 10^−5^	0.01	5	0.03

Shown are regions from the whole-brain ROI-based analyses that demonstrated a significant modulation by salience (top) or by nObservers (bottom) in both experiments after correction for multiple comparisons (using Holm–Bonferroni). *p*-values and *F*-values are presented for the analyses both without covariates (both experiments) and with covariates (*studyforrest* only). Effect sizes (*R*^2^) were calculated based on the model including only the boundary effect (salience/nObservers) as a fixed effect. Region names were taken from the HOA atlas.

### Specificity of hippocampal response to event boundaries

**Figure 4.**
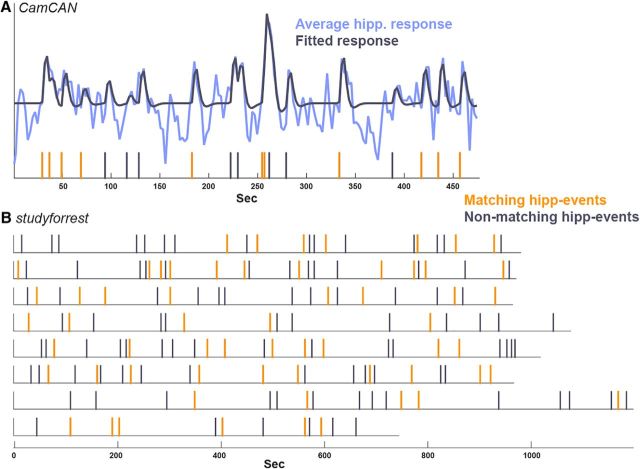
Specificity of hippocampal events (data-driven) to predefined event boundaries. ***A***, Average hippocampal time course in Cam-CAN plotted together with the fitted model. The vertical lines indicate the hippocampal events estimated from the data—the set of events that minimized the residual error of the model when fitting to the hippocampal time course. The model was created by convolving each of these events with an HRF as a separate predictor, yielding the fitted model plotted. The hippocampal events were then divided into those matching a predefined boundary (up to 1 TR from a boundary, in orange, 58% of hippocampal events) and non-matching ones (gray). ***B***, Hippocampal events in each of the eight runs of *studyforrest* divided into matching (38%) and non-matching events.

In the data-driven analysis, we set out to reveal whether increased hippocampal activity is specific to event boundaries. To do so, we defined hippocampal events as points in time that, when modeled as events, best explained the observed hippocampal response, with the number of hippocampal events set according to the number of event boundaries. These hippocampal events were then compared with the predefined boundaries, dividing them into those that matched a predefined boundary (temporal distance of up to 1 TR) versus those that did not (Fig. 4). In Cam-CAN, 11/19 (58%) hippocampal events matched predefined boundaries and, in *studyforrest*, 61/161 (38%) matched. To assess the significance of the match, we compared it with random shuffling of the predefined events, which revealed that both matches were significantly above chance (Cam-CAN: *p* = 0.008; *studyforrest*: *p* < 0.001, with the largest match occurring in the random shuffling being 36, far lower than the actual match of 61).

Together, these complementary analyses suggest the hippocampus is both sensitive and specific in its response to the occurrence of event boundaries. Moreover, its response is modulated by boundary salience even after accounting for shifts in time/location and multiple types of perceptual change.

## Discussion

We examined the relationship between event boundaries in continuous experience and the brain's response measured by fMRI using films as a proxy for real-life experience. In particular, we examined the sensitivity, specificity, and selectivity of the response of the hippocampus given extant but indirect evidence implicating it in processing of event boundaries (Ben-Yakov and Dudai, 2011; Ben-Yakov et al., 2013; DuBrow and Davachi, 2016; Baldassano et al., 2017). In two distinct films, subjective event boundaries were defined by independent observers.

Event boundaries were a reliable trigger for an increased hippocampal response. Moreover, the hippocampal response was sensitive to boundary salience, with the strongest hippocampal response occurring at boundaries identified by the largest proportion of observers. Interestingly, in the longer film (*studyforrest*, which had a sufficient number of events), this sensitivity remained after covarying a large number of measures of perceptual change at those event boundaries. To address specificity, we took an alternative, data-driven approach in which we identified moments in which the hippocampus exhibited the strongest responses and tested the correspondence between these hippocampal events and the subjective event boundaries. In both films, there was a significant match, reaching 58% in Cam-CAN.

Our finding that the hippocampus is sensitive to subjective event boundaries complements other studies that explicitly manipulated boundaries using discrete stimuli such as film clips (Ben-Yakov and Dudai, 2011; Ben-Yakov et al., 2014) or sequences of pictures (DuBrow and Davachi, 2014, 2016; Hsieh et al., 2014) and studies identifying hippocampal sensitivity to spatial boundaries (Doeller et al., 2008; Bird et al., 2010; Gupta et al., 2012; McKenzie and Buzsáki, 2016; Lee et al., 2018). However, it is difficult to determine how much of the hippocampal response in these experiments relates to perceptual change at discrete points in time rather than subjective segmentation of continuous stimulation. Milivojevic et al. (2016) examined hippocampal activity in a continuous film, but focused on the representation of the events themselves rather than sensitivity to boundaries. The only prior study, to our knowledge, that examined hippocampal activity to subjective event boundaries during continuous films is that by Baldassano et al. (2017). The investigators found an increase in hippocampal activity that coincided with shifts in cortical patterns (in AG). These cortical pattern shifts also tended to coincide with subjective boundaries, revealing an indirect link between hippocampal activity and event boundaries in naturalistic experience. Here, we provide more direct evidence that hippocampal activity is driven by subjective event boundaries. Indeed, our data do not support the alternative interpretation that the hippocampal response is driven by pattern shifts in cortical regions because we found an increase in hippocampal activity only at those cortical pattern shifts that coincided with an annotated boundary, suggesting that it is the boundaries, not the pattern shifts, that drive hippocampal activity.

Hippocampal activity was not only triggered by a boundary, but was also graded according to the salience of a boundary measured by the level of agreement across observers. This sensitivity to boundary salience did not appear to reflect purely the degree of perceptual change within the film given that we covaried out a large number of measures of visual and auditory change, including responses in early sensory cortices. Adding these as covariates as well as explicit changes in location or time did remove the significant effect of boundary salience in the Cam-CAN film, but not in the *studyforrest* film. Note that we are not claiming that perceptual changes or changes in location/time do not contribute to hippocampal responses, only that they are insufficient to account for the full range of hippocampal response to subjective boundaries: The hippocampus responded at some moments in time not characterized by a large perceptual change, whereas some salient perceptual changes went “unnoticed” by the hippocampus if they were not experienced as a boundary. We discuss below which feature of event boundaries other than perceptual change may constitute the primary driver of the hippocampal response.

Additional regions exhibited sensitivity to boundary salience: the parahippocampal cortex, lingual gyrus, posterior cingulate, and precuneus, all of which are known to respond at event boundaries and the latter three are modulated by grain of segmentation (Zacks et al., 2006; Speer et al., 2007; Magliano and Zacks, 2011), which may be linked to boundary salience (coarser boundaries being more salient).

### Caveats

One caveat with our study is that we cannot tell whether increased boundary salience reflects increased strength, such that boundaries perceived as stronger are more likely to be detected by observers, increased likelihood of identifying a boundary across participants (rather than any difference in strength within participants, or coarser levels of event segmentation that are likely to elicit more agreement. A second caveat is that, whereas the specificity of the data-driven hippocampal events to subjective boundaries was highly significant, the absolute match was less than half of the *studyforrest* events (38%). This lower correspondence relative to the Cam-CAN film could be due to the lower number of participants, rendering peaks in the average hippocampal response more prone to random noise, and/or to a difference in the nature of the films (the boundaries in *studyforrest* tended to be less clearcut due to the narration). An examination of periods of the film around the occurrence of hippocampal events that did not coincide with boundaries did not reveal a clear trigger; investigation of a wider array of films may identify such additional triggers.

### Functional significance

Two main questions arise as to the nature of hippocampal sensitivity to event boundaries: (1) what constitutes a boundary for the hippocampus and (2) what type of hippocampal processing does the boundary-triggered activity reflect? With regard to what defines a boundary, Event Segmentation Theory (Zacks et al., 2007) postulates that boundaries correspond to spikes in prediction error. However, in naturalistic experience, these spikes are typically associated with both increased change and greater uncertainty and it is difficult to disentangle the two (Richmond and Zacks, 2017). Moreover, prediction error can occur for different features of the event (Zwaan et al., 1995; Huff et al., 2014) such as location, time, or action, which may have additive effects on the probability of event segmentation (Zwaan et al., 1995; Zwaan and Radvansky, 1998; Magliano et al., 2001; Zacks et al., 2009; Huff et al., 2014). Magliano et al. (2001) found that changes in time or movement, but not location, were sufficient to induce an event boundary and Magliano et al. (2011) proposed that action discontinuity was the primary driver of segmentation. The types of change that induce segmentation may depend on the nature of the stimulus and, in films specifically, may depend on the types of continuity editing applied (Magliano and Zacks, 2011; Baker and Levin, 2015). Indeed, through bespoke editing rules designed to create a sense of continuity, large changes can go unnoticed (Smith and Henderson, 2008; Smith et al., 2012; Baker and Levin, 2015). Therefore, perhaps hippocampal boundaries are elicited, not by the degree of perceptual discontinuity, but rather by the sense of conceptual discontinuity that they elicit. For example, a character joining/leaving a conversation may constitute a boundary, eliciting a hippocampal response despite little perceptual change, whereas a cut to a visually distinct frame may elicit no response if it is experienced as part of the same event. This is supported by a recent study finding that participants segmented videos depicting the same actions in first- and third-order perspective similarly despite low similarity of visual features across presentation types (Swallow et al., 2018).

The second question pertains to the functional significance of the hippocampal boundary response. Multiple studies have demonstrated that occurrence of boundaries during encoding shapes the subsequent organization of information in long-term memory (Kurby and Zacks, 2008; Radvansky, 2012; Sargent et al., 2013; Heusser et al., 2018). For example, episodic elements occurring within an event are bound together more strongly than those encountered across events (Ezzyat and Davachi, 2011; DuBrow and Davachi, 2013). Although we did not have measures of memory performance in the current study, an intriguing possibility is that hippocampal activity during the event represents the content of that event (Hsieh et al., 2014; Allen et al., 2016; Milivojevic et al., 2016; Terada et al., 2017) and the increased amplitude of that activity at an event boundary reflects registration to long-term memory of a bound representation of the preceding event (Ben-Yakov and Dudai, 2011; Richmond and Zacks, 2017). This is supported by previous evidence that the hippocampus responds more strongly at the offset of (but not during) subsequently remembered versus subsequently forgotten film clips (Ben-Yakov and Dudai, 2011), combined with the role of the hippocampus in episodic binding (Staresina and Davachi, 2009). A parallel is found in retrieval: the hippocampus is involved in retrieval across event boundaries, but not in within-event retrieval (Swallow et al., 2011). Further research will be required to elucidate the exact nature of the hippocampal response, specifically whether it signals the context shift itself, thereby leading to segmentation in long-term memory (Polyn et al., 2009; Dubrow et al., 2017), or drives rapid replay of the preceding event, creating a cohesive representation (Ben-Yakov and Dudai, 2011; Sols et al., 2017).

In summary, there has been growing interest in the neural basis of memory for naturalistic experience. Although less controlled than typical laboratory studies (e.g., in terms of timing), continuous stimuli are closer to real-life memory. Here, we demonstrate that the hippocampus is sensitive and specific to the occurrence of event boundaries while watching films. Therefore, the hippocampus appears to be important for segmenting continuous experience, most likely to transform continuous experience into representations of discrete events for registration into memory.
